# Impact of osteoarthritis aetiology and implant constraint on the risk of secondary patellar resurfacing after total knee arthroplasty: An analysis of German Arthroplasty Registry (EPRD) data

**DOI:** 10.1002/jeo2.70720

**Published:** 2026-04-16

**Authors:** Stephanie Kirschbaum, Yinan Wu, Oliver Melsheimer, Arnd Steinbrück, Alexander Grimberg

**Affiliations:** ^1^ Charité – University Hospital Berlin, Center for Musculoskeletal Surgery Berlin Germany; ^2^ EPRD Deutsche Endoprothesenregister gGmbH Berlin Germany; ^3^ Orthopädisch‐Chirurgisches Kompetenzzentrum Augsburg (OCKA) Augsburg Germany

**Keywords:** level of constraint, posttraumatic osteoarthritis, revision risk, secondary patella resurfacing

## Abstract

**Purpose:**

The benefit of patella resurfacing (PR) in primary total knee arthroplasty (TKA) remains debated. While outcomes appear similar, unsurfaced patellae show higher revision rates. Existing studies are limited by heterogeneous cohorts. This study assessed the risk of secondary PR in relation to the level of constraint and primary procedure complexity (idiopathic vs. posttraumatic osteoarthritis).

**Methods:**

Using registry data, 258,669 primary TKAs without primary PR were analysed. Demographics, implant constraint and subsequent revisions were recorded. The primary endpoint was secondary PR (without additional femoral or tibial implant removal) over a follow‐up period of up to 8 years.

**Results:**

Cruciate‐retaining (CR) designs showed the lowest revision risk (ca. 1%), whereas posterior‐stabilised (PS) designs showed the highest (ca. 2.0–2.5%, hazard ratio = 1.8 [95% confidence interval = 1.66–1.99], *p* < 0.001). The majority of secondary PR occurred within 4 years. No differences were found between idiopathic and posttraumatic groups.

**Conclusions:**

Registry data indicate higher revision risk with PS versus CR designs, regardless of index surgery complexity.

**Level of Evidence:**

Level III.

**Abbreviations**
BMI: body mass index
BVMed: German Medical Technology Association
CCK: condylar‐constrained
CR: cruciate‐retaining
DGOOC: German Society of Orthopaedics and Orthopaedic Surgery
EPRD: German Arthroplasty Registry
HR: hazard ratio
IQTIG: German Institute for Quality Assurance and Transparency in Healthcare
PR: patella resurfacing
PS: posterior stabilised
RH: (rotating) hinge
TKA: total knee arthroplasty

## INTRODUCTION

The management of the patella in primary total knee arthroplasty (TKA) has been debated for over 30 years. Older studies reported inferior outcomes and more anterior knee pain without patella resurfacing (PR) [3, 4, 16, 21, 35], while recent randomised trials and meta‐analyses did not find significant differences in pain or function between resurfaced and non‐resurfaced patellae using modern implants [6, 9, 14, 28, 33]. Registry studies also reported conflicting evidence: while British, German and Australian registry data demonstrated higher revision rates for primary TKA performed without PR [1, 12, 19], the American registry data cannot confirm these findings [5]. However, a major limitation of existing data is the lack of homogeneity concerning the complexity of the primary procedure (e.g., posttraumatic vs. primary osteoarthritis), implant design and level of constraint. Only the Australian arthroplasty registry examined revision rates of cruciate‐retaining (CR) and posterior‐stabilised (PS) TKA depending on primary resurfacing [19, 20]. Both the German and British registries demonstrated higher revision rates for secondary PR in PS‐ than in CR‐design of some brands [1, 12, 13, 23]. Notably, CR designs account for the majority of TKA procedures for primary osteoarthritis in these countries (59%–77%), which leads to the suggestion that PS designs might be used for the more complex posttraumatic cases and therefore demonstrate higher rates of secondary PR [29]. Therefore, the aim of the present study is to analyse the rates of secondary PR in primary TKA, with a specific focus on (1) the complexity of the index procedure and (2) the level of implant constraint.

## MATERIALS AND METHODS

### Data source

This study utilised data from the German Arthroplasty Registry (Endoprothesenregister Deutschland, EPRD), which has been collecting information on TKAs since November 2012. The EPRD is a non‐profit initiative founded by orthopaedic surgeons and the German Society of Orthopaedics and Orthopaedic Surgery (DGOOC), in collaboration with public health insurers (AOK‐Bundesverband GbR, Verband der Ersatzkassen e.V. [vdek]), the German Medical Technology Association (BVMed), and hospitals performing hip and knee replacements. As of 2023, approximately 78% of all relevant procedures are voluntarily reported to the registry, based on comparisons with data from the German Institute for Quality Assurance and Transparency in Healthcare (IQTIG). The two participating insurance providers (AOK‐B and vdek) cover around 65% of the German population, enabling robust cross‐validation with healthcare claims data. This linkage minimises loss to follow‐up for revision surgeries among patients meeting the inclusion criteria. Ethical approval from the University of Kiel (D 473/11) was obtained.

All primary TKA performed for idiopathic or posttraumatic osteoarthritis of the knee (International Classification of Diseases (ICD) codes M.170, M.171, M.173 and M.174) using CR, PS, condylar‐constrained (CCK) and (rotating) hinged (RH) designs were analysed from the German Arthroplasty Registry (EPRD) database. TKA designs that met the criteria for more than one of the aforementioned categories (e.g., PS‐plus or medial‐pivot designs) were excluded from this study due to their currently limited clinical use. Patients without primary PR were used for further analysis: The primary endpoint was secondary PR (without additional femoral or tibial implant removal) over a follow‐up period of up to 8 years. Implant constraint levels were categorised based on manufacturer information into three groups: CR, PS, CCK and RH. Due to the low frequency of CCK and (rotating) hinged implants in primary TKA procedures, these two implant designs were combined into the category ‘CCKRH’.

### Data analyses

Descriptive data were presented. Continuous variables were reported as median with interquartile range (Q1–Q3), while categorical variables were expressed as absolute numbers and percentages (%). Patient characteristics, including age, sex, body mass index (BMI), Elixhauser comorbidity score and level of constraint, were investigated. Continuous variables were compared using the Wilcoxon rank‐sum test, and categorical variables were compared using Pearson's chi‐squared test. Kaplan–Meier estimates with log‐log 95% confidence intervals (CIs) were used to determine the cumulative revision rate for secondary patellar replacement. Adjusted Cox regression models were applied to calculate hazard ratios (HRs) between CR‐ and PS‐design TKA concerning the risk of secondary PR. Group differences were assessed using the log‐rank test, with statistical significance set at *p* < 0.05. All statistical analyses were performed using R (version 4.4.2; R Foundation for Statistical Computing).

## RESULTS

Overall, 258,669 patients received a TKA for either idiopathic (*n* = 252,189, 97.5%) or posttraumatic (*n* = 6480, 2.5%) osteoarthritis without primary PR. 2120 patients (0.82%) received secondary PR during follow‐up. Male gender was associated with a slightly lower HR for secondary PR compared with female gender. No other patient characteristics were identified as significantly increasing the risk of secondary PR during the follow‐up period (Table 1). Patient characteristics between idiopathic and posttraumatic group as well as distribution of implant design (level of constraint) are shown in Table 2.

**Table 1:** Results of a Cox proportional hazards regression analysing patient characteristics in the overall population with respect to the risk of secondary PR.

Patient characteristic	HR	95% Confidence interval	Significance
Age	0.97	0.96–0.97	<0.001
Male sex	0.9	0.82–0.99	0.036
BMI (compared to normal BMI)			
Underweight [<18.5]	1.47	0.47–4.60	0.5
Pre‐obese [25.0–29.99]	1.09	0.91–1.31	0.3
Obese 1 [30.0–34.99]	1.11	0.92–1.34	0.3
Obese 2 [35.0–39.99]	1.09	0.88–1.35	0.4
Obese 3 [≥40]	0.99	0.78–1.27	0.9
Elixhauser score (compared to healthy patient with score −1)			
0–3	1.02	0.92–1.15	0.7
4	1.14	0.99–1.32	0.064

Abbreviations: BMI, body mass index; HR, hazard ratio; PR, patella resurfacing.

**Table 2:** Demographics and level of constraint in the idiopathic and posttraumatic groups.

	Posttraumatic TKA without primary PR (*n* = 6480)	Idiopathic TKA without primary PR (*n* = 252,189)	Significance
Age	62 (Q1:55, Q3:71)	71 (Q1:63, Q3:77)	<0.001
Sex			<0.001
Female	2911 (45%)	165,786 (66%)	
Male	3569 (55%)	86,403 (34%)	
BMI			<0.001
Underweight [<18.5]	20 (0.3%)	362 (0.1%)	
Normal [18.5–24.99]	997 (15%)	26,875 (11%)	
Pre‐obese [25.0–29.99]	1943 (30%)	65,432 (26%)	
Obese 1 [30.0–34.99]	1288 (20%)	55,470 (22%)	
Obese 2 [35.0–39.99]	518 (8.0%)	27,882 (11%)	
Obese 3 [≥40]	234 (3.6%)	15,047 (6.0%)	
Missing	1480 (23%)	61,121 (24%)	
Elixhauser score			<0.001
(…, −1)	1305 (20%)	57,029 (23%)	
(0–3)	4048 (62%)	142,336 (56%)	
(4, …)	1127 (17%)	52,824 (21%)	
Type of arthroplasty			<0.001
CR	2885 (45%)	163,940 (65%)	
PS	2435 (38%)	75,898 (30%)	
CCKRH	1160 (18%)	12,351 (4.9%)	
Rate of secondary PR			
Received secondary PR	69 (1.1%)	2051 (0.8%)	

Abbreviations: BMI, body mass index; CCKRH, combined group of condylar‐constraint and rotating hinge designs; CR, cruciate‐retaining; PR, patella resurfacing; PS, posterior stabilised; TKA, total knee arthroplasty.

### Revision for secondary PR depending on the level of constraint

**Figure 1:**
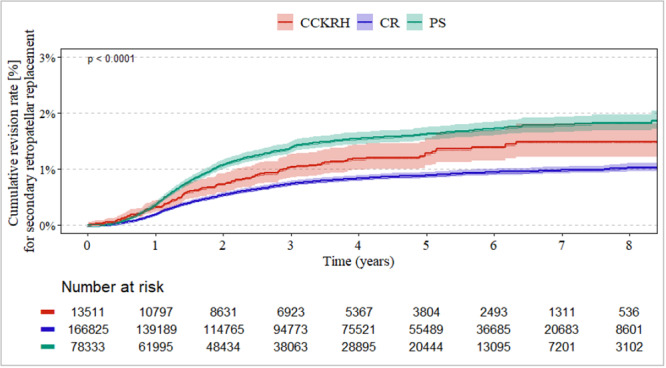
Risk of revision for secondary PR depended on the level of constraint used, independent of the complexity of index surgery (idiopathic and posttraumatic group). CCKRH, combined group of condylar‐constraint and rotating hinge designs; PR, patella resurfacing; PS, posterior stabilised.

Overall risk of revision for secondary PR depended on the level of constraint used (Figure 1, *p* < 0.001). The highest risk for secondary PR was seen in PS designs, followed by CCKRH. CR designs showed the lowest risk for secondary PR over the complete follow‐up time of 9 years. HR for receiving a secondary PR was 1.8 (95% CI = [1.66–1.99], *p* < 0.001) for PS compared to the CR design.

### Revision for secondary PR between the idiopathic and posttraumatic groups

**Figure 2:**
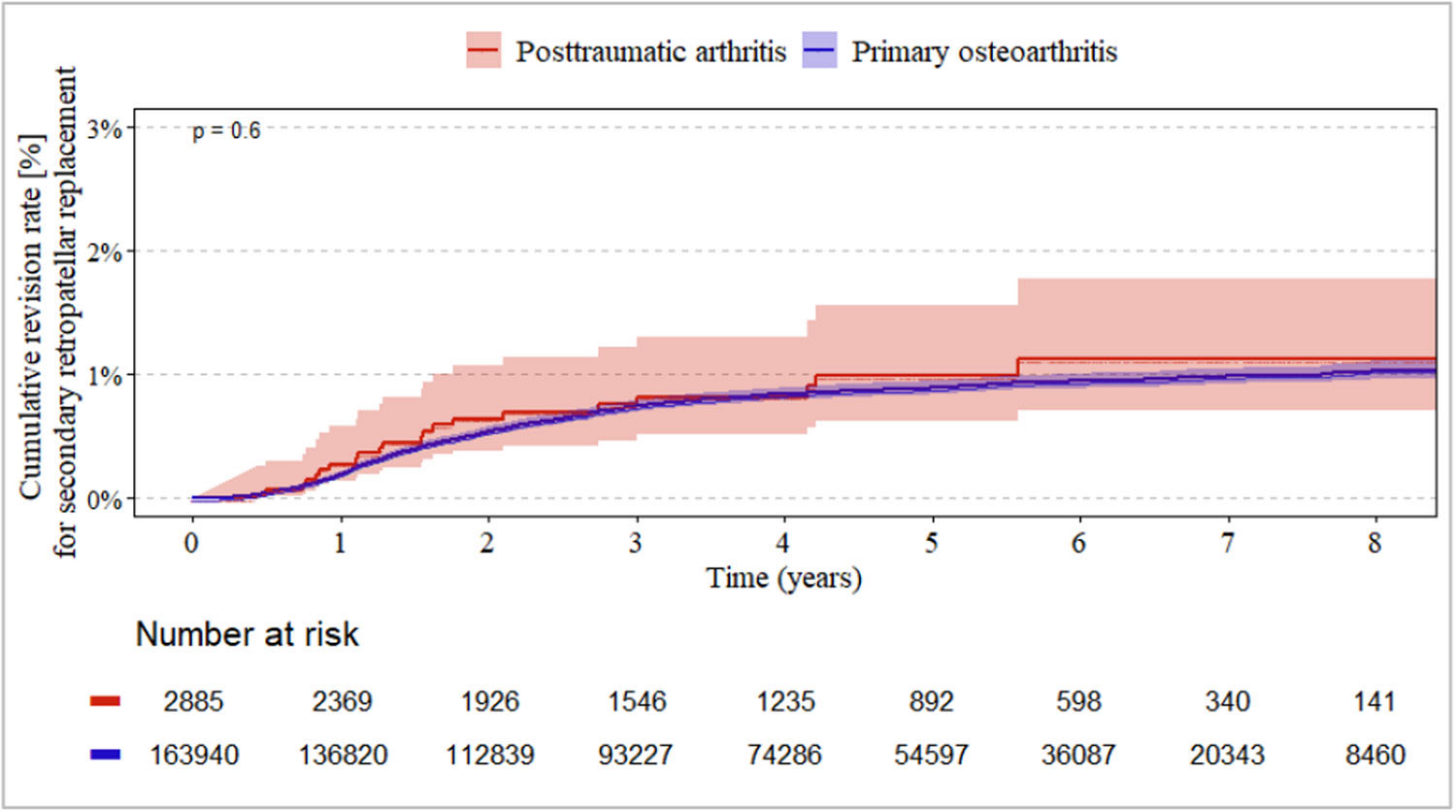
Revision rate for secondary PR between posttraumatic and idiopathic (primary) group in CR‐design implants. CR, cruciate‐retaining; PR, patella resurfacing.

**Figure 3:**
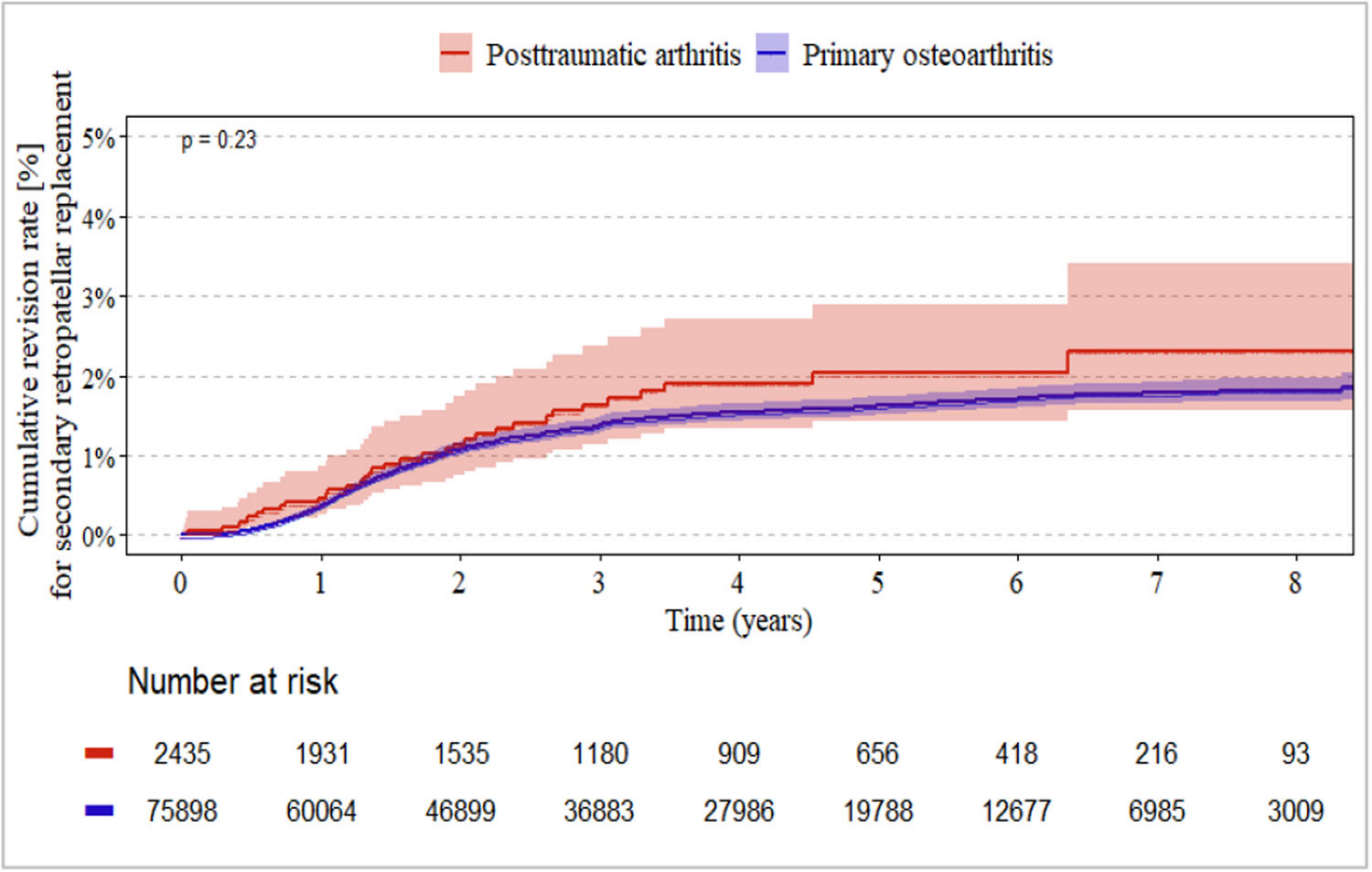
Revision rate for secondary PR between posttraumatic and idiopathic (primary) group in PS‐design implants. PR, patella resurfacing; PS, posterior stabilised.

**Figure 4:**
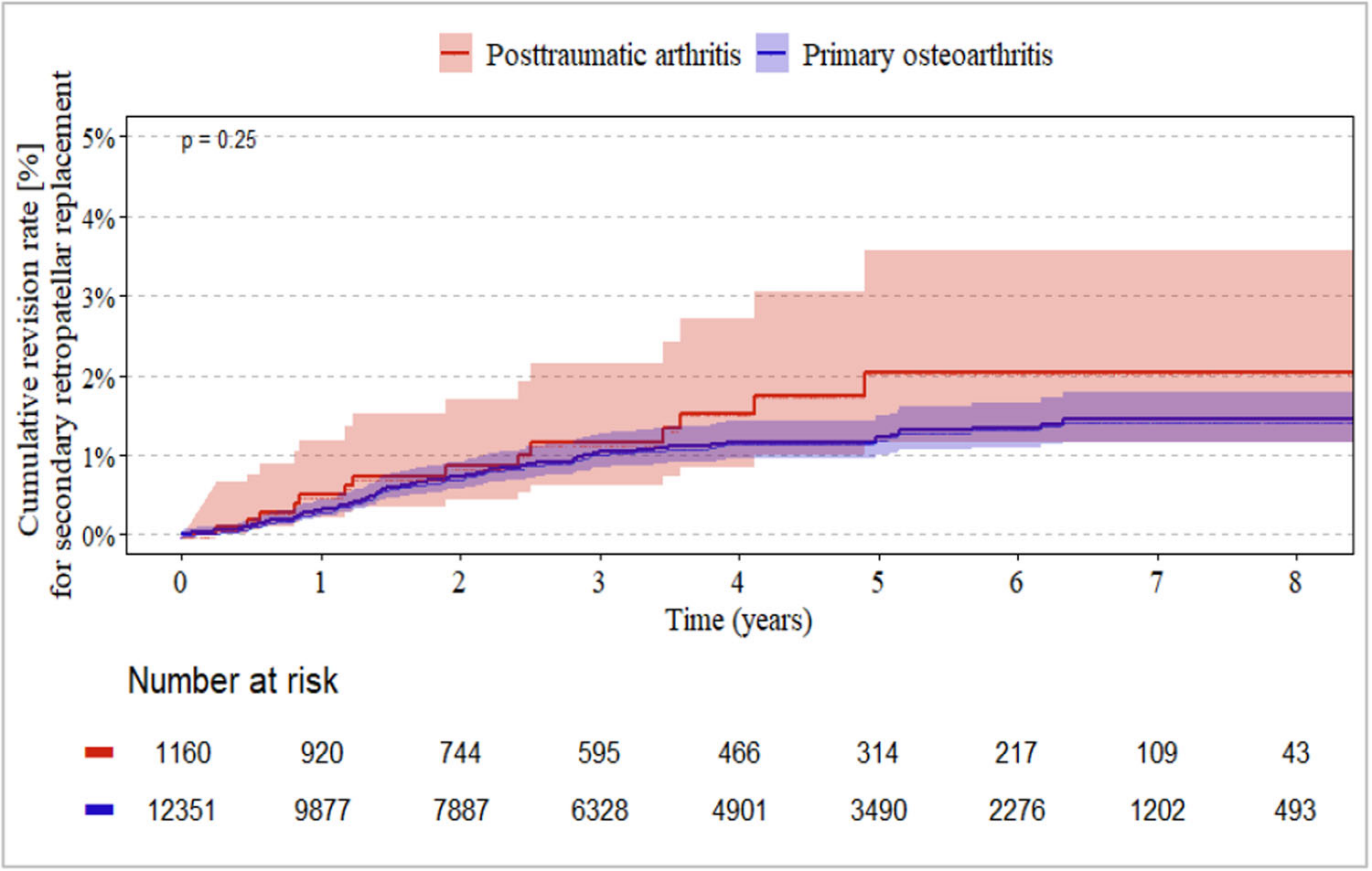
Revision rate for secondary PR between posttraumatic and idiopathic (primary) group in CCKRH‐design implants. CCKRH, combined group of condylar‐constraint and rotating hinge designs; PR, patella resurfacing.

CR designs showed the lowest revision rate for secondary PR (up to 1%, Figure 2) throughout the entire follow‐up period, whereas PS designs had the highest risk (2%–2.5%, Figure 3). There were no significant differences between the idiopathic and posttraumatic groups (each *p* ≥ 0.23). In the constrained group, secondary revision rates for RP ranged between 1% and 2% (Figure 4). Again, there were no significant differences between the idiopathic and posttraumatic groups (*p* = 0.25).

## DISCUSSION

PS‐design showed a twofold higher risk of secondary PR in idiopathic osteoarthritis as well as in more complex posttraumatic situations. Therefore, despite the fact that the complexity of the primary procedure impacts the overall incidence of secondary PR, the level of constraint seems to be an independent risk factor.

The highest secondary PR rates were observed with the PS‐design. The annual report of the EPRD, AOA as well as the NJR indicated that certain PS designs exhibit a higher risk for secondary patellar resurfacing compared to their cruciate‐retaining counterparts when the patella is left unresurfaced [1, 11, 20]. As these countries predominantly use CR‐design TKAs in primary procedures, it is frequently argued that the higher rate of secondary PR or the overall revision rate observed with PS designs is attributable to their more frequent use in more complex cases in these settings. This assumption is typically supported by data from the AJRR, which shows no difference in revision rates between primary unresurfaced and resurfaced patellae when PS designs are predominantly used in primary TKA [5]. Interestingly, comparison between posttraumatic and idiopathic osteoarthritis cases showed no significant difference in the risk of secondary PR among all designs. Therefore, besides the complexity of the case itself, implant design and level of constraint seem to contribute to the risk of secondary PR. Furthermore, the frequently discussed assumption that PS or CCK designs are predominantly used for more complex cases in countries that prefer CR implants—and therefore inflate the incidence of secondary PR as a ‘minor’ revision due to a higher likelihood of persistent pain—could not be confirmed by recent data. Therefore, biomechanical causes should be discussed and examined in future studies.

Possible reasons for the increased risk of secondary PR with PS designs include a potential interaction between the PS‐box and the patella—sometimes also described as patella clunk syndrome—causing potentially anterior knee pain and crepitus with an incidence of up to 12% after TKA [7, 8, 10, 31, 32]. Despite some case series reporting patella clunk syndrome after CR‐design TKA [24], PS‐design seems more prone to patella clunk depending on the intercondylar box ratio and box width [31]. Additional implant‐associated risk factors seem to be a thick anterior flange, increasing the anterior offset as well as a mobile bearing platform [10, 32]. These findings might explain brand‐specific differences concerning susceptibility between CR‐and PS‐versions of the same implant [1, 11]. Besides the potential interaction between the unsurfaced patella and the PS‐box, a reduced patellofemoral contact area in PS compared to CR designs might favour secondary patella erosion or the incidence of anterior knee pain [2]. Another possible biomechanical explanation might be the systematic reduction of the natural slope in the PS design compared to the CR design. Given that the average medial tibial slope reported in the literature ranges between 5° and 11° [17, 27, 30], but PS‐design TKA is recommended to have between 0° and 5° of tibial slope to avoid anterior sliding of the tibial component and anterior impingement of the anterior aspect of the tibial post [25, 26], surgeons typically decrease the individual patient's tibial slope in PS‐design TKA. In different computational simulation studies, a decreased tibial slope on the other hand was associated with increased patellofemoral contact forces in CR‐ and PS‐design TKA [15, 18, 22]. Regarding the interaction of the patella and the PS‐box, as well as altered biomechanics, it seems, therefore, logical that a higher level of constraint causes a more frequent need for secondary PR. Interestingly, the subgroup receiving CCK‐ and hinged designs did not demonstrate a higher risk for secondary PR in the recent study. These findings appear surprising in light of the previously discussed literature and may be explained by the relatively small sample sizes, which could have resulted in underpowered statistical analyses and thus limited the ability to detect a significant overall difference in the CCKRH subgroup. However, the authors' theory is supported when examining brand‐specific evaluations from current registry data. Both the most recent NJR annual report and the latest EPRD report demonstrate, in brand‐specific analyses, substantially higher overall revision risks for CCK and RH designs (1.5‐ to 4‐fold higher revision rates) when patellar resurfacing was not performed [13, 23]. In line with those data, Von Hintze et al. observed increased long‐term revision rates for primary hinged TKAs in the Nordic Arthroplasty Registry when performed without patellar resurfacing [34]. Of course, other confounding factors—such as tibial and femoral component rotation, component alignment, patellar tilt, femorotibial contact point, extent of femoral rollback and trochlear groove design—must also be considered, as they have been shown to influence patellofemoral contact pressures and may therefore contribute to the observed brand‐specific differences [22]. However, despite the observed effect being likely multifactorial, the level of constraint seems to be a significant contributing risk factor. Therefore, primary PR should be carefully considered when using PS‐design TKA.

The present study has some limitations. The results are limited by a low follow‐up for revision rates, currently 8 years. It is possible that the figures could increase even more over a longer follow‐up period. However, as the major difference between the designs seems to manifest within the first 4–5 years, key effects are likely captured within the existing follow‐up timeframe. Second, data for primary CCKRH designs are limited due to the relatively small number of cases, probably resulting in underpowered subgroup analyses. Given the higher level of constraint and the associated biomechanical implications, a higher incidence of patellofemoral complications would be expected in this cohort, consistent with the brand‐specific differences in overall revision rates related to the use of PR reported in current registry data. Extended follow‐up, as well as larger cohorts, may contribute to providing more robust data concerning the isolated risk for secondary PR for this group in future studies.

Differences in alignment strategies may represent another potential source of bias. Kinematic alignment, possibly used in unconstrained TKA designs, is likely to influence patellofemoral contact mechanics differently compared with mechanical alignment, necessarily used in constrained implants. The present registry data do not allow for a detailed assessment of the alignment technique. However, as mechanical alignment remains the predominant approach among TKA surgeons in Germany, this limitation is unlikely to have had a major impact on the current findings. A further limitation is the absence of patient‐reported outcome measures and clinical or radiological assessments. Since the registry is based on coded administrative data, detailed information on preoperative and intraoperative conditions is unavailable. Therefore, confounding risk factors for persisting patellofemoral pain, such as component positioning, cannot be reliably excluded, and secondary PR may be chosen as a ‘minor’ revision to relieve symptoms without addressing the actual underlying cause. However, authors tried to minimise this risk by only choosing isolated secondary PR procedures as the endpoint without any additional exchange of femoral or tibial components for recent analysis. Despite these limitations, the identification of individual contributing risk factors—such as the level of implant constraint but not the complexity of the primary procedure—remains an important goal for current research, to which this study aims to contribute.

## CONCLUSION

Registry data indicate a higher risk of revision for patellofemoral complications following PS design compared to CR design, independent of the complexity of the index surgery.

## AUTHOR CONTRIBUTIONS

**Stephanie Kirschbaum**: Conceptualisation; investigation; validation; writing—original draft; writing—review and editing. **Yinan Wu**: Data curation; formal analysis; investigation; validation; writing—review and editing. **Oliver Melsheimer**: Conceptualisation; data curation; investigation. **Arnd Steinbrück**: Conceptualisation; writing—review and editing. **Alexander Grimberg**: Conceptualisation; data curation; formal analysis; investigation; validation; writing—review and editing.

## CONFLICT OF INTEREST STATEMENT

The authors declare that they received no royalties related to the content of this study. Stephanie Kirschbaum serves as a member of the KSSTA Editorial Board. The remaining authors declare no conflicts of interest.

## ETHICS STATEMENT

Ethical approval from the University of Kiel (D 473/11) had been obtained.

## Data Availability

The data used in this study were obtained from the German Arthroplasty Registry. The data sets are anonymised and not publicly available due to data protection regulations, but may be accessed upon reasonable request and approval by the registry.
